# A surprising finding after adenosine administration

**DOI:** 10.1007/s12471-017-0994-z

**Published:** 2017-04-26

**Authors:** C. Timmermans, H. Wellens

**Affiliations:** 1 Department of Cardiology, Maastricht University Medical Center, Maastricht, The Netherlands; 2 21 Henric van Veldekeplein, Maastricht, The Netherlands

## Answer

**Fig. 1:**
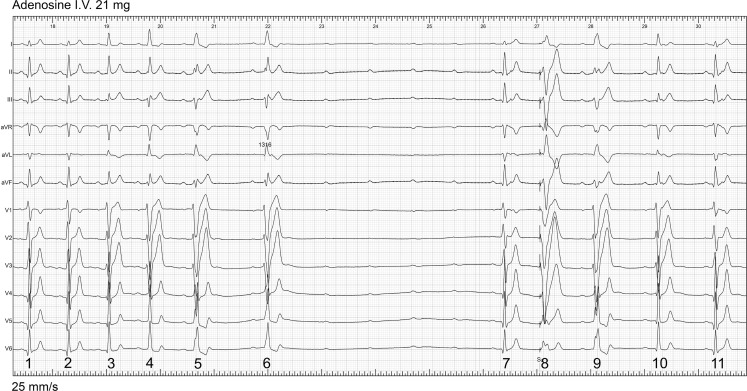
Twelve-lead ECG after adenosine administration during sinus rhythm

To facilitate the explanation of Fig. 1, the QRS complexes are labelled. The first two show a normal QRS preceded by a normal PR interval. Thereafter the QRS configuration changes. Complexes 3 to 6 show gradual widening, a frontal axis shift to horizontal and loss of the initial q in leads I and V6, an rS pattern in lead III, and the development of a left bundle branch block-like QRS configuration. There is also a gradual lengthening of the PR interval. These adenosine-induced changes can be explained by progressive delay and block in AV nodal conduction and progressive activation of the ventricle over a right-sided atriofascicular (AF) fibre, also known as a Mahaim fibre. This structure inserts in the ventricle in or close to the right bundle branch, ultimately resulting in the typical QRS configuration of beats 5 and 6 [1]. Then 4 P waves are not conducted to the ventricle because of complete block both in the AV node and the AF fibre. As described several years ago, in contrast to rapidly conducting accessory pathways, the AF fibre is sensitive to adenosine resulting in delay and block [2]. It is explained by its AV nodal-like cellular composition. In our patient, after adenosine administration during sinus rhythm, complete AV nodal block preceded block in the AF fibre. A similar observation was reported by Belhassen et al. [3]. Of interest is also what happened after the episode of complete AV block. QRS 7 shows AV nodal conduction. QRS 8 is a paced beat from the apex of the right ventricle, with QRS 9 showing anterograde AV conduction only over the AF fibre, indicating retrograde invasion into the normal conduction system. QRS 10 shows AV nodal conduction again with a small contribution to ventricular activation over the AF fibre. QRS 11 is similar to QRS 1 and 2, and has a normal PR, suggesting complete ventricular activation over the AV node. The recording in Fig. 1 clearly shows the short-lasting effect of intravenous adenosine on conduction over the two AV connections. In the patient, tachycardias with anterograde AV conduction over the AF fibre could be initiated by appropriately timed atrial premature beats. Conduction over the AF fibre was terminated by catheter ablation.
